# Guillain-Barré Syndrome and Adjuvanted Pandemic Influenza A (H1N1) 2009 Vaccines: A Multinational Self-Controlled Case Series in Europe

**DOI:** 10.1371/journal.pone.0082222

**Published:** 2014-01-03

**Authors:** Silvana Romio, Daniel Weibel, Jeanne P. Dieleman, Henning K. Olberg, Corinne S. de Vries, Cormac Sammon, Nick Andrews, Henrik Svanström, Ditte Mølgaard-Nielsen, Anders Hviid, Maryse Lapeyre-Mestre, Agnès Sommet, Christel Saussier, Anne Castot, Harald Heijbel, Lisen Arnheim-Dahlström, Par Sparen, Mees Mosseveld, Martijn Schuemie, Nicoline van der Maas, Bart C. Jacobs, Tuija Leino, Terhi Kilpi, Jann Storsaeter, Kari Johansen, Piotr Kramarz, Jan Bonhoeffer, Miriam C. J. M. Sturkenboom

**Affiliations:** 1 Department of Medical Informatics, Erasmus University Medical Center, Rotterdam, The Netherlands; 2 Department of Neurology, Haukeland University Hospital, Bergen, Norway; 3 Department of Pharmacy & Pharmacology, University of Bath, Bath, United Kingdom; 4 Health Protection Agency, London, United Kingdom; 5 Department of Epidemiology Research, Statens Serum Institut, Copenhagen, Denmark; 6 Department of Clinical Pharmacology, Toulouse University Hospital and Institut national de la santé et de la recherche médicale, Toulouse, France; 7 French National Agency for Medicines and Health Products Safety, Saint Denis, France; 8 Medical Doctor - Consultant, Paris, France; 9 Department of Vaccinology, Swedish Institute for Infectious Disease Control, Solna, Sweden; 10 Department Medical Epidemiology and Biostatistics, Karolinska Institute, Stockholm, Sweden; 11 National Institute for Public Health and the Environment, Bilthoven, The Netherlands; 12 Departments of Neurology and Immunology, Erasmus University Medical Center, Rotterdam, The Netherlands; 13 Department of Vaccination and Immune Protection, National Institute for Health and Welfare, Helsinki, Finland; 14 Department of Vaccines, Norwegian Institute of Public Health, Oslo, Norway; 15 Surveillance and Response Support Unit, European Centre for Disease Prevention and Control, ECDC, Stockholm, Sweden; 16 Brighton Collaboration Foundation, Basel, Switzerland; 17 Department of Infectious Diseases and Vaccinology, University Children's Hospital, Basel, Switzerland; Mount Sinai School of Medicine, United States of America

## Abstract

**Background:**

The risk of Guillain-Barré syndrome (GBS) following the United States' 1976 swine flu vaccination campaign in the USA led to enhanced active surveillance during the pandemic influenza (A(H1N1)pdm09) immunization campaign. This study aimed to estimate the risk of GBS following influenza A(H1N1)pdm09 vaccination.

**Methods:**

A self-controlled case series (SCCS) analysis was performed in Denmark, Finland, France, Netherlands, Norway, Sweden, and the United Kingdom. Information was collected according to a common protocol and standardised procedures. Cases classified at levels 1–4a of the Brighton Collaboration case definition were included. The risk window was 42 days starting the day after vaccination. Conditional Poisson regression and pooled random effects models estimated adjusted relative incidences (RI). Pseudo likelihood and vaccinated-only methods addressed the potential contraindication for vaccination following GBS.

**Results:**

Three hundred and three (303) GBS and Miller Fisher syndrome cases were included. Ninety-nine (99) were exposed to A(H1N1)pdm09 vaccination, which was most frequently adjuvanted (Pandemrix and Focetria). The unadjusted pooled RI for A(H1N1)pdm09 vaccination and GBS was 3.5 (95% Confidence Interval (CI): 2.2–5.5), based on all countries. This lowered to 2.0 (95% CI: 1.2–3.1) after adjustment for calendartime and to 1.9 (95% CI: 1.1–3.2) when we accounted for contra-indications. In a subset (Netherlands, Norway, and United Kingdom) we further adjusted for other confounders and there the RI decreased from 1.7 (adjusted for calendar month) to 1.4 (95% CI: 0.7–2.8), which is the main finding.

**Conclusion:**

This study illustrates the potential of conducting European collaborative vaccine safety studies. The main, fully adjusted analysis, showed that the RI of GBS was not significantly elevated after influenza A(H1N1)pdm09 vaccination (RI = 1.4 (95% CI: 0.7–2.8). Based on the upper limits of the pooled estimate we can rule out with 95% certainty that the number of excess GBS cases after influenza A(H1N1)pdm09 vaccination would be more than 3 per million vaccinated.

## Introduction

During the influenza A (H1N1) 2009 pandemic, new monovalent adjuvanted and non-adjuvanted influenza A(H1N1)pdm09 vaccines were introduced in Europe. Immunogenicity and safety was in line with the “Committee for medicinal products for human use (CHMP) Note for Guidance”, but safety data were limited [1]–[3]. Vaccination campaigns started in autumn 2009 at the peak of the pandemic in Europe.

A key safety concern identified in planning the pandemic vaccination campaigns was the potential association between Guillain-Barré syndrome (GBS) and influenza vaccines; this concern stemmed from an association observed in the USA in 1976 between swine flu vaccination and GBS [4]. Subsequent prospective surveillance studies and retrospective epidemiological studies on seasonal influenza vaccines used in 1978, 1992, 1993, and beyond showed no or modest increases in the risk of GBS [5]–[8]. Despite this, the US Food and Drug Administration (FDA), the World Health Organization (WHO) and the European Medicines Agency (EMA) recommended active monitoring of a potential association between the influenza A(H1N1)pdm09 vaccine and GBS.

In Europe, GBS primarily presents as an acute inflammatory demyelinating polyradiculoneuropathy (AIDP) [9]. Three to ten per cent of GBS patients die and an estimated 20% experience continued disability for more than six months [10]. Prospective studies in developed countries have estimated an incidence rate of 2 per 100,000 population per year with an increased risk with age and in males [11]. GBS is thought to be primarily triggered by a preceding respiratory or gastrointestinal infection [12].

The European Centre for Disease prevention and Control (ECDC) commissioned the VAESCO (Vaccine Adverse Events Surveillance and Communication) consortium to study the potential association between influenza A(H1N1)pdm09 vaccine and GBS. A case control study was conducted for a rapid initial assessment with a large-scale more extensive prospective SCCS study carried out in parallel. The VAESCO case control study was based on 104 cases in five European countries and showed no association between A(H1N1)pdm09 vaccine (mostly adjuvanted with AS03) and GBS [13]. In this paper we present the results from the VAESCO SCCS study which included three times the amount of cases.

## Methods

### Setting and design

The VAESCO consortium conducted a prospective self-controlled case series (SCCS) study to investigate the association between influenza A(H1N1)pdm09 vaccination and GBS. A SCCS is a case-only study comparing the incidence of disease during risk and non-risk periods within the same person, inherently controlling for measured and unmeasured confounding factors that remain stable over time [14].

The VAESCO consortium was initiated and core funded by ECDC with the aim of improving post licensure vaccine safety in Europe. It is coordinated by the Brighton Collaboration Foundation and includes partners from public health organizations, regulatory authorities and academic research institutions in Europe.

Centers from Denmark (DK), Finland (FI), France (FR), Netherlands (NL), Norway (NO), Sweden (SE), and the United Kingdom (UK) contributed to the study. All centers used a common protocol and applied the standardised Brighton Collaboration GBS case definition for case classification [9]. Implementation of the protocol and data collection differed per country based on ethical requirements and the healthcare structure. Data harmonization, transformation, and pooling used methods and tools derived from the EU-ADR (Exploring and Understanding Adverse Drug Reactions) project [15]. Centers created harmonized input files according to well-defined instructions. These data files were generated directly from automated resources or manually using customized electronic case report forms. The harmonized input files were transformed using a standardized JAVA-based program (Jerboa® version 2.6.0, September 2010, Erasmus University Medical Center, Rotterdam, Netherlands). Only anonymous and aggregated de-identified information without dates of disease or exposure were shared for individual patient level data pooling and centralised analysis. Consent forms, original data and Jerboa input files were retained at the local centers. Quality control and verification of transmitted data was done at the central data management and analysis center (Erasmus University) in close collaboration with the other centers. All centers commented on the data and results prior to release.

### Source and study population

The total source population exceeded 50 million (M) subjects, with most countries recruiting cases on a national level (NO (4.4 M), SE (9 M), FI (5.5 M), DK (5 M), NL (16 M)). In the UK, the General Practice Research Database (GPRD) (5 M) was used and in France specialized hospitals with a large but undefined catchment area participated. Case recruitment started on 1st November 2009 and lasted maximally until 1st November 2010.

The study population encompassed all cases with GBS or its variant Miller Fisher syndrome with onset of disease during the study period.

Case recruitment-procedures are described in Table 1. Completeness of recruitment was verified retrospectively at the end of the study period by comparing recruited cases with diagnosed case lists (see Table 1). Additional cases identified in this way were included retrospectively where possible. For each subject, follow-up started at the beginning of the study period or date of birth if born after the start of the study period. Follow-up ended with the end of the study period or death occurring prior to the end of the study period.

**Table 1:** Sources of cases, exposure and covariate information per country.

	Cases recruitment	Exposure Information	Covariates during follow-up	Potential bias
**DK**	Cases were identified from the National Patient Register using primary discharge diagnoses only (ICD-10: G61.0). Case validation based on retrospective chart review.	Vaccination registry	None (only from case hospital charts)	Cases: not all charts availableNo ability to control for time varying confounders
**FI**	From hospital Discharge and hospital outpatient records, primary diagnoses (ICD-10 G61.0). Case validation based on retrospective chart review	Vaccination registry	None (only from case hospital charts)	Cases: not all charts availableNo ability to control for time varying confounders
**FR**	Cases were identified prospectively through neurologists in 7 reference hospitals in FR. Patients needed to provide informed consent. Completeness was verified against pharmacy data (immunoglobulin prescriptions) and showed incomplete reporting (<50%), Vaccination status of non-reported cases could not be verified since linkage to vaccination registry required consent.	Ad hoc A(H1N1)pdm09 vaccination registry	Hospital charts and interview, only for period prior to GBS	Incompleteness and potential selection bias cannot be excluded.No ability to control for time varying confounders
**NL**	Cases were identified prospectively through neurologists. Completeness was verified retrospectively by checking against the claims codes in each of the reporting hospitals. Missing patients were included retrospectively in hospitals that were reporting at least one case prospectively.	GP medical record	GP medical record	Small potential for misclassification of exposure since A(H1N1)pdm09 vaccination could also be provided through public health agency for parents of young children
**NO**	Nationwide neurologist reporting network, group of neurologists. Case validation based on review of GBS experts	Vaccination registry	Neurologists, Hospitals, and GPs	Potential selection due to incompletenessInformation on co-variates collected differently for period prior to GBS.
**SE**	Cases of GBS were identified through seven neurology assessment labs where GBS cases are laboratory confirmed for a population of 9.4 million. Informed consent needed to be obtained from all cases. Completeness of cases was checked in the National Patient Registry for part of the country. Recruitment was incomplete because of delays in consent and non-consent. It was not possible to assess whether this non-response differed by vaccination status and hence selection bias cannot be excluded.	By interview at end of follow-up, recall bias cannot be excluded.	By interview for cases at the end of follow up. change in region over time. Should not be used for adjustment	Consent required, potential selection bias.Recall bias (differential recall over time)
**UK**	Each case was identified in the General Practice Research Database by using appropriate READ codes (F370.00, F370000, F370100, F370200, F370z00). Case verification was done using any hospital letters, discharge summaries and GPs' notes recorded as free text. No major selection to be expected	Automated GP records, no recall bias. Non-differential misclassification possible since some persons might have been vaccinated outside of GP office.	GP records	Misclassification of cases due to lack of information on test results

The earliest date of onset of neurological symptoms was the index date. If the date of first symptoms could not be retrieved the date of diagnosis or hospitalization was used. Informed consent was required in SE and FR. Case characteristics were obtained from neurologists or from discharge letters and used to classify cases according to the Brighton Collaboration GBS Case Classification using the Automated Brighton Classification (ABC) tool (www.brightoncollaboration.org).

### Vaccine Exposure

The primary exposure of interest was vaccination with adjuvanted or non-adjuvanted A(H1N1)pdm09 vaccine as recorded in vaccination registries (FR, DK, FI, NO), General Practitioners' (GP) records (NL, UK), or patient interview (SE). The risk period began the day after vaccination and ended 42 days later. If two doses were administered, the risk period of the first dose ended when the second dose was administered. Brand specific information was collected for each influenza A(H1N1)pdm09 vaccination.

### Covariates

Information on several time varying risk factors for GBS was collected during follow-up including seasonal influenza vaccination, influenza-like illness (ILI), upper respiratory tract infections (URTI), and gastrointestinal infections (GI). Each of these covariates was assigned a 42-day risk period. The risk period began on day one of onset of ILI, URTI, or GI or of seasonal influenza vaccine receipt and ended 42 days after onset or exposure. Covariate data were not collected in DK and FI. In FR, covariate data were collected from neurologists at case occurrence for the period prior to GBS only, whereas in SE data on covariates were collected by interview at the end of follow-up. In the UK, NL, and NO general practitioner records were used to collect information on covariates throughout the follow up period; NO also assessed covariates reported by neurologists at the time of case data collection, leading to a potential for differential data collection over time. To adjust for seasonal effects, changes in circulation of the wild type influenza A(H1N1)pdm09 virus and differences in case inclusion over the observation period we considered calendar month as a time varying covariate.

### Statistical Analysis

The RI for the association between A(H1N1)pdm09 vaccine and GBS was estimated using a conditional Poisson regression analysis. This was done for each country separately. Adjustment for calendar month was possible in all countries, whereas further adjustment for ILI, URTI, GI, and seasonal influenza vaccination was only possible in NL, UK, and NO. Sensitivity analyses were used to assess the effects of misclassification of exposure and confounding. An analysis using vaccinated cases only and an analysis using the pseudo-likelihood approach explored confounding by contra-indication to influenza A(H1N1)pdm09 vaccination [14]. A sub analysis was done to assess the impact of residual confounding by ILI, URTI, seasonal influenza vaccination, and GI infections. Misclassification of the risk period was investigated by applying risk periods smaller than 42 days. In order to study effect modification by infections occurring just prior to GBS onset, stratified analyses were carried out for age, sex, history of GBS, and prior infections (ILI, URTI, GI) in UK, NL, and NO. The country specific estimates were pooled applying a random effects model. All analysis used SAS v9.1 (Cary, North Carolina).

## Results

**Figure 1:**
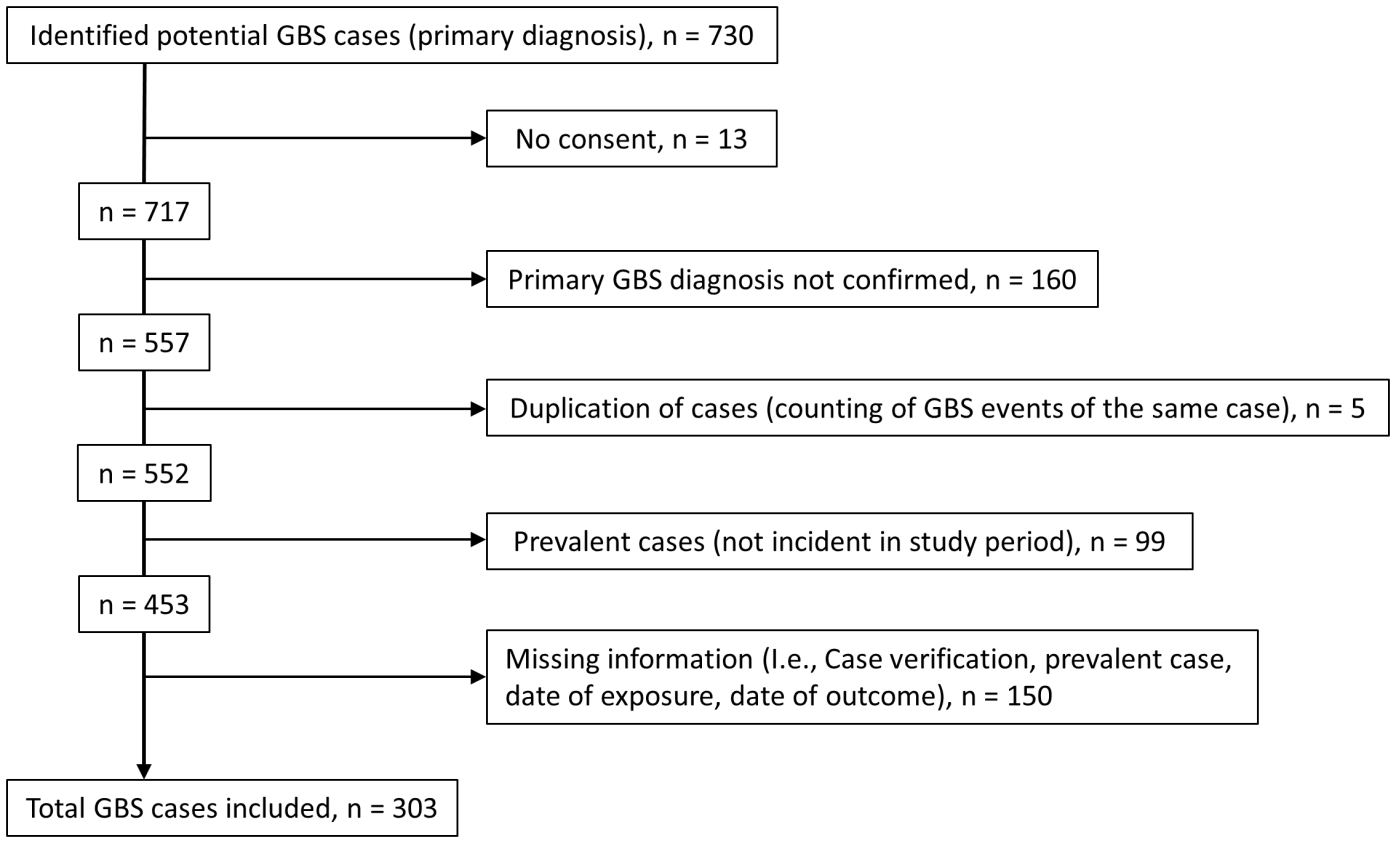
Flowchart of case inclusion.

**Figure 2:**
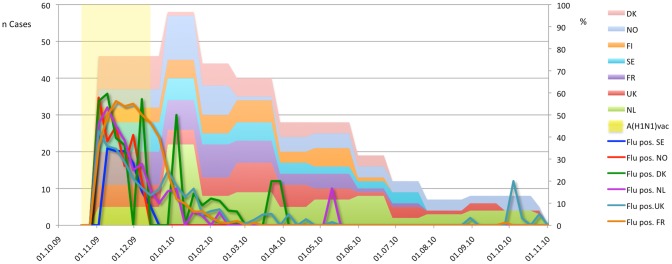
Inclusion of GBS cases (DK, FI, FR, NL, NO, SE, UK), influenza A(H1N1)pdm09 immunization period (influenza A(H1N1)vac), and percentage of flu positive cases among all tested per country (Flu pos. DK, …, Flu pos. UK; Source: ECDC 2011) over total study period.

**Figure 3:**
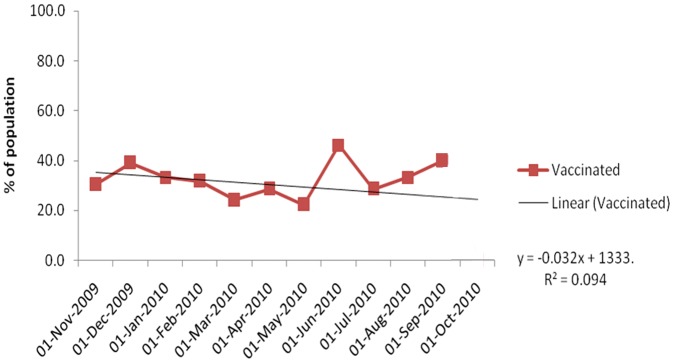
Inclusion of vaccinated cases (% of population) over study period.

In total 730 potential GBS cases were identified during the study period. Of these, 427 cases were excluded (see figure 1), leaving 303 GBS cases in the study population. Case inclusion declined over time from 133 cases in the first three months to 18 in the last three months (Figure 2). The percentage of influenza A (H1N1) pdm09 vaccinated cases did not change significantly over time (R^2^ = 0.094; Figure 3).

Cases had a mean age of 50 years (SD: 4.1) ranging from 45 (SD: 20.8) years in the NL to 56 (SD: 19.5) years in NO, less than 10% were younger than 20 years. On average the follow-up period was 321 days. Case classification differed by country, primarily depending on the type of data source used for case recruitment. Of all cases, 36% were classified as Brighton Collaboration level 1, 26% as level 2, 13% as level 3, and 25% as level 4a. In 69 cases electrophysiology (mostly AIDP) had either not been performed for diagnosis or was not recorded. On a scale from 0 to 6, with 0 meaning complete physical fitness and 6 meaning death, the disability score was most frequently 4 (30.6%) (Table 2).

**Table 2:** Characteristics of Guillain-Barré syndrome cases.

Characteristic	DK		FI		FR		NL		NO		SE		UK		Total	
	N	%	N	%	N	%	N	%	N	%	N	%	N	%	n	%
Cases in study period	31	10.2	29	9.6	41	13.5	80	26.4	50	16.5	32	10.6	40	13.2	303	100
Females	14	45.2	12	41.4	20	48.8	32	40.0	25	50.0	12	37.5	17	42.5	132	43.6
**Mean age (SD)** 1 **[years]**	49.2 (20.2)		54.4 (20.8)		50.0 (21.9)		45.0 (20.8)		55.5 (19.5)		51.5 (20.2)		45.4 (20.4)		50.1 (4.1)	
Age ≤4	0	0.0	0	0.0	1	2.4	2	2.5	1	2.0	0	0.0	2	5.0	6	2.0
Age 5–19 years	3	9.7	3	10.3	4	9.8	10	12.5	0	0.0	2	6.3	3	7.5	25	8.3
Age 20–59 years	18	58.1	10	34.5	18	43.9	44	55.0	21	42.0	15	46.9	24	60.0	15	49.5
Age ≥60	10	32.3	16	55.2	18	43.9	24	30.0	28	56.0	15	46.9	11	27.5	122	40.3
**Brighton Classification** 2																
1	10	32.3	17	58.6	13	31.7	28	35.0	21	42.0	19	59.4	0	0.0	108	35.6
2	8	25.8	3	10.3	16	39.0	30	37.5	14	28.0	8	25.0	0	0.0	79	26.1
3	4	12.9	7	24.1	7	17.1	11	13.8	5	10.0	5	15.6	0	0.0	39	12.9
4a	9	29.0	2	6.9	5	12.2	10	12.5	10	20.0	0	0.0	40	100.0	76	25.1
Unknown	0	0.0	0	0.0	0	0.0	1	1.3	0	0.0	0	0.0	0	0.0	1	0.3
**Electrophysiology**																
AIDP^3^	23	74.2	16	55.2	15	36.6	36	45.0	29	58.0	23	71.9	0	0.0	142	46.9
AMAN^4^	0	0.0	0	0.0	1	2.4	6	7.5	3	6.0	0	0.0	0	0.0	10	3.3
AMSAN^5^	1	3.2	2	6.9	0	0.0	4	5.0	1	2.0	6	18.8	2	5.0	16	5.3
Equivocal	0	0.0	0	0.0	6	14.6	9	11.3	7	14.0	1	3.1	0	0.0	23	7.6
Normal	2	6.5	0	0.0	2	4.9	3	3.8	6	12.0	0	0.0	0	0.0	13	4.3
Not performed	5	16.1	11	37.9	12	29.3	20	25.0	3	6.0	2	6.3	0	0.0	53	17.5
Unresponsive nerves	0	0.0	0	0.0	0	0.0	1	1.3	1	2.0	0	0.0	0	0.0	2	0.7
Unknown	0	0.0	0	0.0	5	12.2	1	1.3	0	0.0	0	0.0	38	95.0	44	14.5
**GBS disability score** 6																
0	0	0	0	0.0	6	14.6	0	0.0	0	0.0	0	0.0	0	0	6	2.0
1	0	0	0	0.0	0	0.0	5	6.3	7	14.0	1	3.1	0	0	13	4.3
2	0	0	9	31.0	2	4.9	19	23.8	11	22.0	6	18.6	0	0	47	15.5
3	0	0	4	13.8	10	24.4	21	26.3	6	12.0	7	21.9	0	0	48	15.8
4	0	0	13	44.8	11	26.8	20	25.0	17	34.0	10	31.3	0	0	71	23.4
5	0	0	2	6.9	2	4.9	13	16.3	8	16.0	7	21.9	0	0	32	10.6
6	0	0	1	3.4	0	0.0	1	1.3	1	2.0	1	3.1	0	0	4	1.3
Unknown	31	100.0	0	0.0	10	24.4	1	1.3	0	0.0	0	0.0	40	100.0	82	27.1
**Index month**																
Nov 2009	9	29.0	4	13.8	9	22.0	5	6.3	5	10.0	8	25.0	6	15.0	46	15.2
Dec 2009	1	3.2	5	17.2	8	19.5	22	27.5	12	24.0	6	18.8	4	10.0	58	19.1
Jan 2010	6	19.4	5	17.2	9	22.0	8	10.0	8	16.0	3	9.4	5	12.5	44	14.5
Feb 2010	5	16.1	6	20.7	6	14.6	9	11.3	1	2.0	5	15.6	8	20.0	40	13.2
Mar 2010	4	12.9	3	10.3	3	7.3	5	6.3	4	8.0	3	9.4	6	15.0	28	9.2
Apr 2010	3	9.7	5	17.2	4	9.8	7	8.8	4	8.0	2	6.3	3	7.5	28	9.2
May 2010	3	9.7	1	3.4	1	2.4	8	10.0	3	6.0	2	6.3	1	2.5	19	6.3
Jun 2010	0	0.0	0	0.0	1	2.4	2	2.5	3	6.0	3	9.4	3	7.5	12	4.0
Jul 2010	0	0.0	0	0.0	0	0.0	3	3.8	3	6.0	0	0.0	1	2.5	7	2.3
Aug 2010	0	0.0	0	0.0	0	0.0	4	5.0	2	4.0	0	0.0	2	5.0	8	2.6
Sep 2010	0	0.0	0	0.0	0	0.0	4	5.0	4	8.0	0	0.0	0	0.0	8	2.6
Oct 2010	0	0.0	0	0.0	0	0.0	3	3.8	1	2.0	0	0.0	1	2.5	5	1.7
TOTAL	31		29		41		80		50		32		40		303	

^1^ Standard Deviation.

^2^ Sejvar J. J. et al. 2011, Guillain-Barre syndrome and Fisher syndrome: case definitions and guidelines for collection, analysis, and presentation of immunization safety data. Vaccine 29(3).

^3^ AIDP: acute inflammatory demyelinating polyradiculoneuropathy.

^4^ AMAN: acute motor axonal neuropathy.

^5^ AMSAN: acute motor and sensory axonal neuropathy.

^6^ Current disability score at the time of case assessment and/or inclusion into the study.

Abbreviations: DK: Denmark; FI: Finland; FR: France; NL: Netherlands; NO: Norway; SE: Sweden; UK: United Kingdom; GBS: Guillain-Barré syndrome.

Overall, 99 cases (33%) received influenza A(H1N1)pdm09 vaccination, mostly adjuvanted with AS03, before symptom onset (Table 3). Of these, 36 (37%) cases developed GBS within 42 days after a first dose of influenza A(H1N1)pdm09 vaccination whereas 7 cases occurred within the exposure risk window but after a second dose of influenza A(H1N1)pdm09 vaccination.

**Table 3:** Guillain-Barré syndrome occurrence during follow-up and during the 6-week (42 days) risk periods following influenza A(H1N1)pdm09 vaccination and infection.

Exposure	DK		FI		FR		NL		NO		SE		UK		TOTAL	
	n	%	n	%	n	%	n	%	n	%	n	%	n	%	n	%
**Cases in study period**	31	10.2	29	9.6	41	13.5	80	26.4	50	16.5	32	10.6	40	13.2	303	100
**Follow-up total (mean) in days**	11,286 (364.1)		6,127 (211.3)		11,421 (278.6)		26,322 (329)		17,845 (356.9)		11,471 (358.5)		12,666 (316.7)		97,138 (320.6)	
**Cases exposed to influenza A(H1N1)pdm09 vaccine anytime during follow-up period**	4	12.9	13	44.8	5	12.2	29	36.3	23	46.0	22	68.8	3	7.5	99	32.7
**GBS in influenza A(H1N1)pdm09 vaccination risk period**																
1^st^ dose1)	2	6.5	4	13.8	2	4.9	10	12.5	8	16.0	9	28.1	1	2.5	36	11.9
2^nd^ dose1)	0	0.0	0	0.0	2	4.9	5	6.3	0	0.0	0	0.0	0	0	7	2.3
**Cases during risk period following seasonal influenza vaccination**	0	0	1	3.4	1	2.4	4	5	4	8	0	0	5	12.5	15	0.05
**Cases during risk period following infections (ILI, URTI) risk period**	6	19.4	6	20.7	10	24.4	16	20	30	60.0	8	25.0	3	7.5	79	26.11
ILI1)	5	16.1	1	3.4	2	4.9	6	7.5	13	26.0	1	3.1	1	2.5	29	9.6
URTI1)	1	3.2	5	17.2	8	19.5	10	12.5	17	34.0	7	21.9	2	5	50	16.5

^1)^% per number of cases included per country.

Abbreviations: ILI: influenza like illness; URTI: Upper respiratory tract infection; UK: United Kingdom; NL: Netherlands; FR: France; SE: Sweden; FI: Finland, NO: Norway, DK: Denmark.

Few countries could collect data on time-varying covariates over the entire follow-up period. Most countries assessed covariates at the time of case collection, but not afterwards, and therefore these data could not be utilized for adjustments but could be used for stratification. Based on the information collected at case occurrence, 15 cases developed GBS within 42 days after seasonal influenza vaccination and 79 cases developed GBS within 42 days after onset of ILI or URTI (Table 3).

### Risk ratio of GBS

The crude country specific RI of GBS during the influenza A(H1N1)pdm09 vaccination risk period compared to the non-risk period varied from a low of 1.6 in FI to a high of 7.7 in DK (based on two exposed cases only), with an overall pooled estimate of 3.5 (95% CI: 2.2 to 5.5). Adjustment for calendar month had a significant impact (RI: 2.0, 95% CI: 1.2 to 3.1). Sensitivity analyses accounting for contra-indication after GBS onset showed a minor change in the calendar month adjusted pooled RI from 2.0 to 1.9 (95% CI: 1.1 to 3.2) when the pseudolikelihood method was used, and 1.8 (95% CI: 0.7 to 4.7) when considering vaccinated cases only (Table 4).

**Table 4:** Relative incidence estimates for the association between infections, influenza A(H1N1)pdm09 vaccination, seasonal influenza vaccination and Guillain-Barré syndrome.

	DK		FI		FR		NL		NO		SE		UK		Pooled	
	RI	95% CI	RI	95% CI	RI	95% CI	RI	95% CI	RI	95% CI	RI	95% CI	RI	95% CI	RI	95% CI
**Covariates**																
ILI	NA		NA		NA		10.5	3.0–36.3	30.6	8.6–108	NA		1.8	0.2–16.0	10.4	2.6–41.1
URTI	NA		NA		NA		13.0	4.3–39.2	17.7	6.2–34.7	NA		2.2	0.4–10.6	8.51	3.0–24.0
GI	NA		NA		NA		11.6	2.8–49.4	53.31	6.56–433	NA		2.3	0.2–22.6	11.9	2.5–55.6
Seasonal influenza vaccination	NA		NA		NA		1.2	0.4–4.0	5.5	1.6–18.9	NA		6.0	1.8–19.7	3.9	1.8–8.3
**Any influenza A(H1N1)pdm09 vaccination**																
Unadjusted	7.7	1.1–54.4	1.6	0.5–5.4	6.4	1.0–40.4	2.7	1.3–5.9	3.9	1.6–9.3	4.8	2.1–11.1	3.3	0.3–36.5	3.5	2.2–5.5
Adjusted for calendar month	3.9	0.5–32.2	1.6	0.5–5.4	2.9	0.4–19.6	1.4	0.6–3.4	1.9	0.7–5.2	2.7	1.0–7.8	2.3	0.2–27.7	2.0	1.2–3.1
**Adjustment effect any influenza A(H1N1)pdm09 vaccination in NL, NO, UK**																
Adjusted for calendar month only							1.4	0.6–3.4	1.9	0.7–5.2			2.3	0.2–27.7	1.7	0.8–3.4
Fully adjusted (month, ILI/URTI, GI)							1.2	0.5–3.3	1.5	0.5–4.6			1.5	0.1–23.1	1.4	0.7–2.8
**Sensitivity analysis on influenza A(H1N1)pdm09 vaccination for contra-indication**																
**Pseudolikelihood**																
1st dose	3.6	0.4–29.5	3.2	0.7–14.6	0.6	0.1–6.7	1.3	0.4–4.0	1.6	0.6–4.3	2.4	0.8–6.9	4.8	0.3–83.9	1.9	1.1–3.2
2nd dose	NA		NA		2.2	0.2–26.3	1.2	0.4–3.4	NA		NA		NA		1.3	0.5–3.4
**Vaccinated cases only**	NE		2.6	0.2–32.5	NE		1.2	0.2–8.3	1.6	0.3–7.9	2.5	0.4–16.0	NE		1.8	0.7–4.7

Abbreviations: NA: not available or not valid; NE = Not estimable due to small numbers or absence RI: relative incidence; ILI: influenza like illness; URTI: upper respiratory tract infection, GI: gastrointestinal Infection, UK: United Kingdom; NL: Netherlands; FR: France; SE: Sweden; FI: Finland, NO: Norway, DK: Denmark.

In NL, NO, and the UK where further adjustment for infections, seasonal influenza vaccination, and other time dependent covariates was possible, the RI for the association between influenza A(H1N1)pdm09 vaccination and GBS decreased from the unadjusted pooled RI of 3.2 (95% CI: 1.8 to 5.6) to 1.7 (95% CI: 0.8 to 3.4) after adjustment for calendar month, and to 1.4 (95% CI: 0.7 to 2.8) upon further adjustment for ILI, URTI, and GI.

Sensitivity analyses using different post-exposure risk periods resulted in a calendar month-adjusted pooled RI of 2.3 (95% CI: 1.4 to 3.8) for the first four weeks. The RI was 2.3 (95% CI: 1.2 to 4.4) in the first two weeks and 2.6 (95% CI 1.4 to 4.9) during weeks three to four.

We did not observe statistically significant interactions between age, infections, or seasonal influenza vaccination and the association between the influenza A(H1N1)pdm09 vaccination and GBS (Table 5).

**Table 5:** Stratified analyses for association between influenza A(H1N1)pdm09 vaccination and Guillain-Barré Syndrome.

	DK		FI		FR		NL		NO		SE		UK		Pooled (random effects)	
	RI	95% CI	RI	95% CI	RI	95% CI	RI	95% CI	RI	95% CI	RI	95% CI	RI	95% CI	RI	95% CI
**Changing risk windows**																
1–28 days	4.4	(0.5 to 35.6)	1.0	(0.2–4.6)	1.3	(0.1–12.6)	2.5	(1.0–6.4)	2.2	(0.8–6.1)	2.7	(0.9–7.8)	4.2	(0.4–50.2)	2.3^4)^	(1.4–3.8)
1–14 days	7.6	(0.9–61.7)	2.3	(0.5–10.6)	3.4	(0.3–33.3)	2.5	(0.7–9.3)	1.3	(0.3–5.9)	1.0	(0.2–4.7)	10.8	(0.9–133.2)	2.3^5)^	(1.2–4.4)
15–28 days	NE^3)^		0.0		0.0		1.9	(0.7–5.5)	2.5	(0.8–7.8)	3.7	(1.2–11.1)			2.6	(1.4–4.9)
**42 day risk window**																
19–59 years old	0.0		3.3	(0.5–19.3)			1.0	(0.1–10.7)	0.6	(0.1–5.5)	1.0	(0.2–6.6)			1.3	(0.5–3.6)
older than 59 years	2.3	(0.1–38.0)	0.0		0.0		1.1	(0.3–4.9)	3.5	(1.0–12.6)	7.6	(1.6–35.8)	11.9	(0.4–365.5)	3.2	(1.5–6.9)
Co-morbidities1)	0.0		2.5	(0.2–35.5)	0.24		0.0		3.2	(0.6–17.0)	0.0				3.0	(0.7–12.3)
No co-morbidities1)	0.0		1.7	(0.4–6.7)	1.7	(0.1–19.6)	1.9	(0.6–6.6)	1.4	(0.4–5.3)	0.0				1.7	(0.8–3.4)
Seasonal influenza vacvination	0.0		3.0	(0.2–50.4)	0.2		0.5	(0.1–3.6)	2.1	(0.2–19.0)	0.0				1.2	(0.3–4.5)
No seasonal influenza vaccination	0.0		1.6	(0.4–6.4)	4.8	(0.3–83.6)	2.2	(0.4–11.2)	1.7	(0.6–5.4)	0.0				1.9	(0.9–4)
ILI, URTI infection	NE		1.1	(0.1–10.6)	2.9	(0.2–51.9)	1.1	(0.1–11.4)	1.4	(0.4–4.8)	3.2	(0.8–14.0)			1.8	(0.8–3.9)
No ILI, URTI infection	2.5	(0.2–34.4)	2.2	(0.5–10.3)	0.0		1.5	(0.4–5.8)	3.6	(0.5–24.3)	2.7	(0.6–13.2)			2.2	(1.1–4.7)

1) Malignancy, immune suppression, or autoimmune disorder NE = Not estimable due-small numbers.

Abbreviations: RI, relative incidence; ILI, influenza like illness; URTI, Upper respiratory tract infection; UK, United Kingdom; NL, Netherlands; FR, France; SE, Sweden; FI, Finland, NO, Norway, DK, Denmark.

## Discussion

Based on a source population of more than 25 million subjects from NL, UK, and NO we found no significant elevated association between the risk of GBS following immunization with an adjuvanted influenza A(H1N1)pdm09 vaccine, when adjusted for all known measurable confounders (RI 1.4, 95% CI: 0.7 to 2.8). This result is very similar to that of the VAESCO consortium case control study, published previously using one third of the cases from fewer countries [13]. In DK, FI, FR and SE we could not adjust for time varying confounders such as infections since data were not collected over the entire follow up period. Pooling data from all seven countries yielded a crude RI of 3.5, which reduced to 2.0 (95% CI: 1.2 to 3.1) after adjustment for calendar month: this pooled estimate still comprises residual confounding by infections. The effect of calendar month may be explained by it being a good proxy for circulation of the wild-type influenza A(H1N1)pdm09 virus (see figure 2).

This study is unique as it directly pools data on individual patients from seven European countries, using a common protocol, common case definition, common infrastructure, and common data elaboration. The impact of methodological issues that occurred due to differences in implementation of the protocol could be assessed by comparing the association accross countries; the consistency observed is reassuring. Beyond the effect of the influenza A(H1N1)pdm09 vaccination on GBS this study underlines the advantages of collaborative transnational vaccine safety studies. They not only increase the scale of a study, but also allow for consistency- checks across sources in the absence of bias from differences in design and methods. The use of common methods and subsequent pooling reaches far beyond the traditional approach of meta-analyses where rather heterogeneous estimates resulting from different designs, methods, and settings are being pooled.

The data from this VAESCO study are in line with other results from Europe with studies from FR (RI 0.9, 95% CI: 0.1 to 7.6) [16], SE (RI 1.1 95% CI: 0.6 to 1.9) [17], and the UK (RI 1.05, 95% CI: 0.37–2.24) [18], all showing no association. In contrast, a recent report from Germany, where AS03 adjuvanted vaccine was used, showed an increased risk of GBS after vaccination (RI 4.65, 95% CI: 2.17 to 9.98) [19]. German investigators had already started a separate SCCS study and thus elected not to participate in VAESCO. They did not adjust for infections or calendar-time and selection bias could not be excluded since cases originated from a reporting network. Pooling of calendarmonth adjusted RI estimates with the VAESCO study would be possible through meta-analysis to enlarge the scale of the current EU based study. Five studies from the US, where non-adjuvanted influenza A(H1N1)pdm09 vaccines were used, have recently been published. Each of the initial observational studies found an increased RI ranging from 1.6 (95% CI: 1.0 to 2.2) [20], to 2.1 (95% CI: 1.2 to 3.5) [21], to 2.5 (95% CI: 0.42 to 15.0) [22], and to 4.4 (95% CI: 1.3 to 14.2) [23]. Three studies used self-controlled designs but without further adjusting for time-varying confounders [21]–[23]. The study assessing the lowest RI (1.6 (95% CI: 1.0 to 2.2)) was a cohort study adjusting for age and sex [20]. The highest RI of 4.4 (95% CI: 1.3 to 14.2) was based on data from the US Vaccine Safety Datalink (VSD) project, which was based on 13 vaccinated cases [23]. Salmon et al. recently published a meta-analysis of US studies on the association between influenza A(H1N1)pdm09 vaccines including two unpublished studies and reported a pooled estimate of 2.35 (95%CI: 1.42–4.01) [24]. A SCCS study from Quebec, Canada adjusted for seasonality and contraindication using vaccinated cases only reported a relative risk of 1.9 (95% CI: 1.0 to 3.5) [25]. After the first VSD study, a second VSD study was recently published, investigating specifically the effect of antecedent infections on the relative incidence of GBS following influenza A(H1N1)pdm09 vaccines, using a case centered analysis. This analysis showed the impact of infections as a confounding factor [26]. After adjusting for antecedent infections, there was no evidence for an elevated GBS risk following 2009–10 monovalent/2010–11 trivalent influenza vaccines. However, the association between GBS and antecedent infection was strongly elevated. The effect of infections on the risk of GBS and the potential preventive effect of vaccination on the risk of GBS by preventing influenza was recently discussed by Stowe and Poland [27], [28]. This recent evidence underlines the need to adjust for infections as we could do in part of the countries in our analyses.

Owing to its observational nature, our study suffers from limitations that should be considered when interpreting data. In NL and SE, where reporting networks were used, completeness of recruitment was verified by retrospectively comparing included cases with claims made for GBS. In FR and SE informed consent was required which could be another reason for non-inclusion. Finally, since cases were included only if charts/medical records could be reviewed, lack of data could be another source of selection bias. The distribution of vaccinated cases over time showed no significant trend, suggesting changes in the number of cases included over time were not related to exposure and selection bias may be limited (Figure 3).

Information bias may arise from misclassification of the outcome as well as the exposure. Cases recruited directly from neurologists (i.e., FR, NL, NO, and SE) generally had higher levels of diagnostic certainty. In the UK all cases were classified with the lowest Brighton Collaboration case certainty level as information was retrieved retrospectively from GP medical records, which capture information from specialist letters but often lack information on specific test results. In DK cases were classified based on retrospective review of specialist charts resulting in partially missing information. As standardized criteria were used for case classification, misclassification of the outcome will be minimal. In all countries prospectively collected health care records were used to obtain information on exposure, except in SE, which relied on interviews and may have suffered from recall bias. In the NL exposure may have been misclassified in young children (<5 years) who were participating in mass vaccination campaigns, but this will be non-differential and there were very few paediatric cases. Exposure might be misclassified due to misspecification of the risk period. Sensitivity analysis showed no difference in the RI when the risk window was restricted to 15 to 28 days after vaccination (RI 2.6, 95% CI: 1.4 to 4.9); compared to the first two weeks (RI 2.3, 95% CI 1.2 to 4.4) and the risk in a 4-week risk window (RI 2.3, 95% CI 1.4 to 3.8).

We addressed confounding both by design (SCCS controls for time-constant confounders), through adjustments, and sensitivity analyses. GBS could be a contra-indication for influenza A(H1N1)pdm09 vaccine as a similar vaccine had been associated with GBS in the past. To investigate this issue we carried out analyses including only vaccinated subjects and analyses applying the pseudo-likelihood method [14]. The pseudo-likelihood method reduced the calendar-adjusted pooled RI from 2.0 to 1.9 and if only vaccinated cases were included to 1.8, indicating that contra-indications were a minor issue. Calendar month acted as an important confounding factor, not because time itself is a risk factor, but because it may serve as a proxy for influenza A(H1N1)pdm09 circulation, which was highly time-dependent and co-occuring with the mass vaccination campaigns (see figure 2). Adjustment for additional timevarying confounders (mainly infections) lowered the pooled calendar-month adjusted RI in NL, NO, and UK from 1.7 to 1.4. This is in line with the effect of control for infections seen by Greene et al [26]. The effect of infections on the risk of GBS differed substantially between countries due to differences in timing and type of data collection methods. In future studies, standardization of covariate exposure reporting will have to be addressed in more detail. Given the variation in the RI of GBS among other countries' A(H1N1)pdm09 vaccinees, these results, as well as the pooled estimate that was adjusted for calendar month only, are likely affected by residual confounding by infections.

## Conclusion

This large, multinational SCCS study confirms the results from the initial much smaller VAESCO case control study. In each country, the unadjusted association between influenza A(H1N1)pdm09 vaccine and GBS suggests a possible increase in risk, and adjustment for confounders consistenly lowered this risk. Further adjustment for infections could only be carried out in some countries and demonstrated the effect of confounding by ILI, GI and URTI, which themselves were strong risk factors for GBS. After adjustment we did not observe an association between influenza A(H1N1)pdm09 vaccine and GBS. Based on the upper limit of the confidence interval of both the partially and fully adjusted RI estimates we can rule out with 95% certainty that adjuvanted influenza A(H1N1)pdm09 vaccines (mainly AS03 adjuvanted) would have resulted in more than 2 or 3 excess cases of GBS per 1 million vaccinated persons.
